# Olfactory assessment in treatment and survivor groups of pediatric solid malignancies

**DOI:** 10.1007/s12672-026-05012-1

**Published:** 2026-04-16

**Authors:** Yasin Yilmaz, Deniz Tugcu, Betul Eda Cilesiz, Fatma Ezgi Dogan, Vildan Kocali, Gulcan Erbas, Osman Kuleli, Mustafa Bilici, Sifa Sahin, Isil Vatansever, Comert Sen, Gulsah Tanyildiz, Serap Karaman, Aysegul Unuvar, Zeynep Karakas, Richard L. Doty

**Affiliations:** 1 Department of Pediatrics, Division of Pediatric Hematology and Oncology, Istanbul University Istanbul Medical Faculty, Millet Caddesi, Topkapı, Fatih, 34098 İstanbul, Turkey; 2 Department of Otorhinolaryngology, Istanbul University Istanbul Medical Faculty, Istanbul, Turkey; 3 Perelman School of Medicine, Smell and Taste Center, Philadelphia, USA

**Keywords:** Cancer, Children, Olfactory dysfunction, Hyposmia, Pediatric smell wheel

## Abstract

**Background/aim:**

Children with cancer who have received or are receiving chemotherapy and radiotherapy are at risk of serious effects of their treatments. Although most of these problems are well documented, the same cannot be said for the sense of smell. We aimed to determine whether there is a decrease in smell function in children with solid tumor malignancies who are currently undergoing or are survivors, using a specially designed, validated quantitative smell test for children.

**Materials and methods:**

Sixty children with solid tumor (31 post-treatment and 29 current treatment) and 25 healthy controls were administered the Pediatric Smell Wheel^®^. Health-related quality of life (QOL) was assessed using the Pediatric Quality of Life Inventory (PedsQL).

**Results:**

The cancer study group had significantly lower PSW scores than the control group [respective means (95% CIs) = 8.85 (8.43;9.17) and 10.32 (9.91;10.73); *p* < 0.001]. Twenty-two (36%) had scores less than 9, indicating the presence of olfactory dysfunction. PSW scores did not differ between patients with or without surgery and with or without radiotherapy (respective ps = 0.51 and 0.94). A weak positive correlation between PSW scores and the self-ratings of smell ability was present (*r* = 0.28, *p* = 0.02). Although lower QOL scores were found for the study group than for the controls for each domain (*p* < 0.01), such scores were not significantly correlated with those of the PSW.

**Conclusion:**

This study demonstrates that treatments for solid tumors of a variety of types significantly impact the ability of children to smell. The real-life implications of such deficits are yet to be determined.

## Introduction

Approximately 400,000 children are diagnosed with cancer every year worldwide [1]. In the United States, 15,000 children are diagnosed each year [2]. The most common types of cancer seen in childhood are leukemias, brain tumors, lymphomas and other solid tumors such as Wilms’ tumor, neuroblastoma, rhabdomyosarcoma and bone tumors In high-income countries, where integrated services are generally available, five-year survival rates exceed 80% for children with cancer, but are less than 30% in low- and middle-income countries (LMICs) [3].

Acute and chronic complications of cancer treatments are closely followed up in comprehensive clinics. Children with cancer who have received chemotherapy and radiotherapy are at risk of serious late effects of their treatments, including endocrinopathies, musculoskeletal problems, hearing loss, neurocognitive problems, and heart failure [4]. A long-term follow-up analysis of a cohort of children who were treated and survived cancer forty years ago showed that more than half had experienced a serious or disabling complication by the age of 50 [5]. However, children with cancer treated in recent years have a lower risk of late effects due to changes in treatment regimens and increased endeavors to identify late effects [6].

Vision and hearing problems due to chemotherapy or radiotherapy, in addition to cancer itself, are occasionally encountered and mentioned in the literature. Platinum based agents are particularly prone to cause some degree of hearing loss and new agents have been developed to prevent such loss. Although, complications to vision and hearing are often followed and managed, this is not the case with the sense of smell. To date, only a few studies have examined the olfactory function of children with cancer [7, 8].

Smell receptors have a high turnover rate and are regularly replaced from basal cells. This division mechanism renders these cells sensitive to chemotherapy and radiotherapy. The frequent exposure of cytotoxic therapy to basal cells causes an alteration in the structure of the receptors and basal cells [9]. This may end up with impairment in recovery of chemosensory function [10]. Rates of at least one change in taste or smell function have been reported to be as high as 70% among patients with cancer [10, 11].

In this study, we assessed smell function of children being treated for cancer or who had completed such treatment, along with healthy age-matched controls, using a well-validated standardized smell test specifically designed for children (the Pediatric Smell Wheel^®^) [12]. Moreover, we screened each child’s health-related quality of life using the Pediatric Quality of Life Inventory version 4.0 (PedsQL) [13].

## Materials and methods

### Participants

The cancer-related study group included twenty-seven girls and thirty-three boys [mean (SD) age = 13.3 (4.3) years; range 6–21 years] from the Istanbul Medical Faculty, Pediatric Oncology Clinic. The control group consisted of 12 healthy girls and 13 healthy boys [mean (SD) age = 13.7 (2.0) years; range 7–18 years] who had been admitted to ambulatory pediatrics for routine general examinations.

This study was conducted between September of 2024 and March of 2025. The cancer-related study group included patients who were (a) diagnosed with cancer and were currently undergoing treatment (*n* = 29) or (b) cancer survivors who visited the clinic during the study period (*n* = 31). The inclusion criteria were the diagnosis of cancer (solid tumors such as lymphoma, brain tumor, Wilms tumor, neuroblastoma, Ewing sarcoma, osteosarcoma, rhabdomyosarcoma), eligible age (older than 6 years), and regular follow up at our clinic. The exclusion criteria were leukemia, tumors located in olfactory area and acute infections (i.e., acute rhinitis, allergic rhinitis, sinonasal fungal infection, respiratory tract infection). Patients and parents were informed about the study and informed consent was obtained.

The mean disease duration (time since diagnosis) was 3.0 years and duration since treatment completion for the survivor group was 2.9 years. Diagnoses were Hodgkin lymphoma (23%), Non-Hodgkin lymphoma (20%), central nervous system tumor (17%), Rhabdomyosarcoma (14%), Wilms tumor (8%), Ewing sarcoma (7%), Osteosarcoma (5%), Neuroblastoma (3%) and hepatocellular carcinoma (3%). All patients received chemotherapy while 55% of them underwent radiotherapy and 39% surgery. 35% received radiotherapy to head and neck areas (Table 1).

**Table 1:** Sociodemographic and clinic data of participants

Characteristics	Study Group (*n* = 60)	Healthy Group (*n* = 25)	*p* value
**Gender** (f)	27 (45%)	12 (48%)	
**Age** mean ± SD years (median)	13.3 ± 4.3 (14.0)	13.7 ± 2.8 (14.0)	0.67
**BMI** mean ± SD (median)	19.6 ± 4.5 (19.4)	19.3 ± 4.3 (18.3)	0.76
**Hand of choice** (r)	51 (85%)	24 (96%)	x^2^:2.0, *p* = 0.15
**Disease Duration** mean ± SD years (median)	3.0 ± 3.2 (2.1)		
**Age of Diagnosis** mean ± SD months (median)	123.1 ± 54.3 (128.5)		
**Diagnosis** Hodgkin Lymphoma Non-Hodgkin Lymphoma CNS Tumor Rhabdomyosarcoma Wilms Tumor Ewing Sarcoma Osteosarcoma Neuroblastoma HCC	14 (23%) 12 (20%) 10 (17%) 8 (14%) 5 (8%) 4 (7%) 3 (5%) 2 (3%) 2 (3%)		
**Treatment Options** Chemotherapy Radiotherapy Surgery	60 (100%) 33 (55%) 23 (39%)		
**Status** Undergoing Treatment Survivor	29 (48%) 31 (52%)		
**Types of Chemotherapy** Platin Based Nitrogen Mustard Etoposide Vinca Alkaloid Anti-Metabolites	16 (27%) 34 (57%) 19 (32%) 52 (87%) 16 (27%)		
**Radiotherapy to Head and Neck** Yes	21 (35%)		

### Test procedures

This study was approved by the Institutional Review Board (Istanbul University, Istanbul Medical Faculty Clinical Research Ethics Committee) (No. 2023/1128) in accordance with the Declaration of Helsinki. All participants were informed that they could withdraw from the study at any time, and written informed consent was obtained. Upon acceptance into the study and following signed informed consent, each subject was administered the Pediatric Quality of Life Inventory version 4.0 (PedsQL) [13]. This 23-item questionnaire assesses four health-related quality of life domains: Physical, Emotional, Social, and School Functioning. The psychosocial status is the total of the emotional, social, and school domains. PedsQL was translated into Turkish language and validation studies were performed in 2007 [14]. For each quality-of-life domain tested, item scores were coded 0 (always) to 100 (never) using the standard scoring algorithms and summed within each subgroup.

The Pediatric Smell Wheel^®^ (PSW, Sensonics International, Haddon Hts., NJ, USA) consists of a cardboard wheel or disk that rotates within an outer jacket, such that only one microencapsulated ‘‘scratch and sniff’’ odorant at a time is exposed for sampling [12]. Positioned along the circumference of the wheel are microencapsulated (“scratch and sniff”) labels containing the following odorants – onion, soap, popcorn, bubblegum, banana, cherry, rose, chocolate, smoke, peppermint and cinnamon. Four multiple-choice alternatives consisting of both words and pictures are positioned under each odorant label and the participant is required to provide an answer even if no smell is perceived or the answer is unknown (forced-choice testing). Each answer is signified by filling in a small circle next to the intended response alternative. When the test is completed and the disk completely rotated, the correct answers appear as dark dots in a series of holes in the jacket. The number of holes that have marks signify the total test score, i.e., the number of items that were correctly identified. According to guideline of PSW, a total of 9 or more correct answers is regarded as normosmia in children, 8 or less score indicates olfactory dysfunction. This test was previously performed in the pediatric Turkish population with diabetes and epilepsy [15, 16]. This test was also adapted to other cultures, such as Brazilian population [17].

### Data analysis

Components of the Statistical Package for the Social Sciences (SPSS version 23.0, IBM Corp.) were employed for statistical analyses. Descriptive statistics were used for main definitive characteristics. The Student’s t-test was used to compare means and the chi square test was used for categorical variables. Correlations were performed using the Pearson correlation coefficient. Results were depicted as means with associated 95% confidence intervals; *p* < 0.05 was considered statistically significant.

## Results

### Comparison between study group and healthy group

The study group had lower PSW scores than the control group [respective means (95% CIs) = 8.85 (8.43, 9.17) and 10.32 (9.91, 10.73); *p* < 0.001]. The range for PSW scores was [5–11] for study group. Twenty-two patients (36%) had scores less than 9, indicating the presence of olfactory impairment. Among these patients, 2 were diagnosed with a brain tumor; 10 were diagnosed with lymphoma. The olfactory scores were not correlated with age, disease duration, and BMI in the study group (ps = 0.78, 0.32 and 0.75, respectively). PSW scores did not differ between males and females (*p* = 0.22).

Besides olfactory testing, patients were asked to rate their own ability to smell on a 10-point Likert scale ranging from zero (worst) to ten (best). The mean (SD) score was 8.27 (1.48) (95% CI 7.88;8.65). There was a significant, albeit weak, positive correlation between PSW scores and the self-ratings of smell ability (*r* = 0.28, *p* = 0.02).

Mean Quality of Life (QOL) indices were lower for the study group than for the controls for the domains of Physical Function [72.39 (95% CI 67.42, 78.44) vs. 90.73 (85.33, 96.13), *p* < 0.001], Emotional Function [69.00 (95% CI 64.07, 74.57] vs. 85.40 (77.52, 93.27), *p* = 0.001], Social Function [91.16 (95% CI 87.71, 94.48) vs. 98.60 (97.20, 99.99), *p* < 0.001], and School Function [63.89 (95% CI 58.90, 68.88) vs. 88.40 (83.28, 93.51), *p* < 0.001]. The total QOL questionnaire score was also lower in the study group than in the control group [73.34 (69.36, 77.31) vs. 90.78 (86.85, 94.71), *p* < 0.001) (Table 2). No significant correlations were apparent between PSW scores and scores from each QOL domain (ps > 0.05).

**Table 2:** Olfactory Test Scores and PedsQL scores for study and healthy group

Characteristics [mean (SD)]	Study group (*n* = 60)	Healthy group (*n* = 25)	*p* value
Olfactory test scores	8.85 (1.23)	10.32 (0.98)	< 0.001
**PedsQL domains**			
Physical function	72.39 (21.36)	90.73 (13.08)	< 0.001
Emotional function	69.00 (20.12)	85.40 (19.08)	0.001
Social function	91.16 (12.90)	98.60 (3.39)	< 0.001
School function	63.89 (19.14)	88.40 (12.39)	< 0.001
Psychosocial status	74.66 (13.08)	90.79 (9.42)	< 0.001
Overall health status	73.34 (15.24)	90.78 (9.51)	< 0.001

### Comparison within study group (undergoing treatment vs. survivors)

Within the study group, patients currently undergoing treatment had non-significant lower PSW scores than those of the survivor group [8.59 (95% CI 7.92;9.26) vs. 9.00 (8.62;9.38); *p* = 0.26] (Table 3). 41% of the children (*n* = 12) undergoing treatment had olfactory impairment compared to 32% of the survivors (*n* = 10). PSW scores did not differ between patients with or without surgery and with or without radiotherapy (respective ps = 0.51 and 0.94). The PSW scores did not differ among those receiving different forms of chemotherapy [i.e., with/without platin-based (*p* = 0.52), nitrogen mustard (*p* = 0.56), etoposide (*p* = 0.46), vinca alkaloid (*p* = 0.14) and anti-metabolite chemotherapy (*p* = 0.73)]. The mean PSW scores of those with CNS tumors (*n* = 10), lymphoma (*n* = 26), other solid tumors (*n* = 24) are shown in Table 4.

**Table 3:** Olfactory Test Scores for undergoing treatment and survivor group

Characteristics [mean (SD)]	Undergoing treatment group (*n* = 29)	Survivor group (*n* = 31)	*p* value
Olfactory test scores	8.59 (1.76)	9.00 (1.03)	*p* = 0.26

**Table 4:** Olfactory Test Scores according to diagnosis

Characteristics [mean (SD)]	CNS Tumor (*n* = 10)	Lymphoma (*n* = 26)	Other Solid Tumor (*n* = 24)	*p* value
Olfactory test scores	9.40 (1.26)	8.81 (0.84)	8.54 (1.91)	*p* = 0.28

## Discussion

We found that 36% of 60 children with various forms of cancer, of which 29 (48%) were currently undergoing treatment and 31 (52%) were not (“survivors”), exhibited some degree of smell dysfunction relative to controls. No meaningful differences in test scores between the two cancer-related groups were evident. Olfactory dysfunction was unrelated to disease duration, tumor or cancer type, treatment choices, and chemotherapy options. Moreover, the olfactory scores were not significantly correlated with scores on the quality-of-life scale. However, a significant, albeit weak, correlation was evident between the PSW scores and the patients’ self-ratings of smell ability (*r* = 0.28, *p* = 0.02).

Our results differ from those of an earlier study of survivors of childhood cancer [7].In their cohort of 51 survivors, whose cancer treatments ended ~ 5 years earlier, only six (11.8%) had some degree of smell dysfunction with two (3.9%) being classified as hyposmic, a frequency not different from controls in a previously obtained normative database. While the olfactory dysfunction rate of our study was nearly three times higher for survivors (36%), the time since last treatment was less than 3 years. This fact, along with the slightly higher rate exhibited by those in our study who were currently undergoing cancer treatments (41%), suggests the possibility that long-term improvement in olfactory dysfunction may occur after cessation of cytotoxic treatment. However, this may not be the case in light of a study of 32 childhood medulloblastoma survivors tested for smell function a mean of 20.5 years after their last treatment. In that study, 17 (53%) evidenced hyposmia based upon objective testing [18]. The reasons for such differences are not entirely clear, although the types of cancers involved and differences in olfactory test procedures cannot be ruled out.

The other interesting result of this study was that nearly half of the patients with olfactory dysfunction were diagnosed with lymphoma. We speculated that this finding could be related to therapy, since most of patients with lymphoma received radiotherapy to neck area and this can cause damage to retronasal route of smell function. Another factor might be chemotherapy differences among the cancer patients.

A number of long-term follow-up studies of children with cancer have revealed a number of wide-ranging and variable complications later in life. Thus, childhood cancer survivors are at high risk for physical and psychosocial health effects at later stages of life. Some treatment-related problems can be effectively managed, but others can continue into adulthood [19]. Childhood cancer survivors are twice as likely to report serious chronic health problems by the age 50 than healthy controls, and their mortality rate is 10 times higher than the general population [20]. Comprehensive, well-organized, long-term follow-up (LTFU) care programs are often established by pediatric oncology societies to optimize health outcomes. These programs are implemented in many clinics treating cancer patients [21]. Although hearing and eye problems were very well monitored, we have been unable to find any monitoring of smell problems in LTFU programs. These findings, together with other large cohort studies, illustrate this gap in the literature.

Olfactory dysfunction can have serious effects on daily life, including quality of life, cognitive functions, and diet. Smell is an important sense that regulates appetite, food intake, and the taste of food. Pleasant food aromas can increase appetite and aid food localization. There are two ways for receiving the smell of foods; orthonasal and retronasal pathway. Both the orthonasal and retronasal pathways play a regulatory role in eating behavior [22]. The sense of smell also plays an important role in social communication. Body odors carry various information that guides nonverbal communication, such as familiarity, nutrition, hormonal status, and emotionality. In addition, body odors strongly influence the mother-child bond [23].

The sense of smell is a sensory function that serves as a warning mechanism. The human sense of smell is capable of detecting gaseous hazards at very low concentrations, such as smoke and fire, as well as metabolic products released during food spoilage. These odors typically evoke a feeling of disgust, thereby serving as a warning signal for human health [24]. It also supports certain motor functions such as balance, fine motor skills, respiratory function, and swallowing. Moreover, sense of smell enhances spatial orientation and the development of cognitive maps [25].

Olfactory dysfunction generally has negative outcomes on food consumption and energy balance. This might end up with either loss of interest in eating and in turn weight loss or increasing food consumption to compensate the lack of aroma perception and in turn weight gain [26, 27]. Thus, the loss of smell, can cause a decrease in the quality of life related to food. In addition to food-related problems, emotion processing is also impaired in relation to smell loss. Olfactory dysfunction is associated with depressive and anxiety symptoms [28]. Moreover, the decline in olfaction-related quality of life can increase depressive symptoms [29]. As a result, loss of smell affects quality of life in some way, being associated with psychological distress, depression, anxiety, and loneliness [24, 30].

As in the adult population, olfactory dysfunction is underestimated in daily pediatric clinic routines [31]. More researches among miscellaneous pediatric populations could help raise awareness about the consequences of smell disorders in children’s daily lives and help understand these consequences. These consequences include effects on family relationships, peer connections, and emotional and cognitive functions [24, 32, 33].

Our study has both strengths and weaknesses. One of its strengths is the use of an objective and validated olfactory test specifically designed to engender interest and attention of young people. Although our sample size was modest (*n* = 60), it was comparable with other studies of this topic. Another strength, although it might also be considered a weakness, is that our study population was heterogeneous, albeit all pediatric solid tumor patients. Moreover, the mean time since the end of treatment for the survivor group was relatively short.

In conclusion, a high prevalence of olfactory dysfunction was found in both survivors and children with cancer under treatment. However, children may not aware of their deficit. Health teams should be conscious of these kinds of neglected issues.

## Data Availability

Data supporting the findings of this study are available on request from the corresponding author. The data are not publicly available due to privacy or ethical restrictions.
